# The achievement of pre-operative expectations in patients undergoing knee arthroplasty: a cohort study evaluating unique patient goals

**DOI:** 10.1186/s41687-024-00734-8

**Published:** 2024-06-06

**Authors:** Sascha Karunaratne, Ian Andrew Harris, Mark Horsley, Lyndal Trevena, Michael Solomon

**Affiliations:** 1 Surgical Outcomes Research Centre (SOuRCe), Royal Prince Alfred Hospital (RPAH), PO Box M157, Sydney, NSW 2050 Australia; 2 Institute of Academic Surgery (IAS), Royal Prince Alfred Hospital, Sydney, Australia; 3 Department of Orthopaedic Surgery, Royal Prince Alfred Hospital, Sydney, Australia; 4 School of Public Health, Faculty of Medicine and Health, The University of Sydney, Sydney, Australia; 5 Institute for Musculoskeletal Health, Faculty of Medicine and Health, The University of Sydney, Sydney, Australia

**Keywords:** Patient expectation, Knee arthroplasty, Osteoarthritis, Objectives/Goals

## Abstract

**Background:**

Total knee arthroplasty (TKA) is a common procedure employed to treat end-stage osteoarthritis. While TKA is generally believed to have acceptable outcomes, many patients report pain or functional deficits not in line with their expectation following the procedure. It has been postulated that patient’s pre-operative expectations regarding post-operative treatment outcomes play a significant role in satisfaction. It is therefore important to assess if the outcomes of surgery truly align with patient’s individual expectations. Thus, the purpose of this study was to determine the degree to which patient expectations of TKA are achieved and the contribution of TKA to achieving patient goals one year after surgery.

**Methods:**

A consecutive sample of 110 patients booked for total knee arthroplasty were asked to identify their most important goals to inform the Direct Questioning of Objectives Index (DQO Index, range 0 to 1) and identify their surgical goals and grade their expectation that a knee arthroplasty would achieve each goal on an 11-point scale. One year after surgery, the DQO Index was repeated to assess their current ability to achieve each pre-operative goal, and asked to estimate the contribution of their knee arthroplasty in achieving each goal. Mean differences between baseline and one year follow-up were calculated regarding the DQO Index and expected achievement of pre-operative goals.

**Results:**

According to the DQO Index at one year, patients improved from a poor quality of life pre-operatively (mean ± standard deviation: 0.20 ± 0.18) to moderately high quality of life (mean ± standard deviation: 0.71 ± 0.21) reflecting a large improvement in ability to achieve each goal. Although achievement improved, for each goal, the patient estimates of the extent to which the knee arthroplasty had contributed to achieving the goal was lower than their initial expectation provided pre-operatively (mean difference range: 0.6 to 1.9 on an 11-point scale).

**Conclusion:**

Patients undergoing TKA have high expectations that their surgery will address their primary goals. Despite surgery largely achieving these goals (improved pain and function), the extent to which the goals were achieved was lower than patients had expected pre-operatively.

**Supplementary Information:**

The online version contains supplementary material available at 10.1186/s41687-024-00734-8.

## Introduction

Total knee arthroplasty (TKA) is a common procedure and is employed to treat end-stage osteoarthritis (OA), particularly when patients no longer respond to conservative management [1, 2]. Knee arthroplasty has shown greater improvements in function compared to non-operative treatment, but it is also associated with a higher risk of serious adverse events [3, 4]. While TKA is viewed as a generally acceptable procedure, many patients report pain or functional deficits after the procedure [5, 6]. The associated dissatisfaction with these persistent deficits is a significant issue that has continued to warrant further investigation [5]. Tempering expectations of surgery has been postulated as one of the key factors that may be able to modulate satisfaction post-operatively. However, the exact criteria required to be addressed is difficult to identify given the variation in how individual patients present and their pre-existing notions of what this surgery can offer them [7–9].

Patients are generally optimistic regarding the outcomes of TKA and have expectations that reflect this [10]. It is important to assess if the outcomes of surgery truly align with patient’s individual expectations. The expectations of patients have been generally found to consist of the improvement of pain, function and quality life [7–10]. However, the specific nature of how patients develop these expectations and whether they are fulfilled is less clear. This greatly influences if a patient can make the best decision for their unique needs, where they are able to be adequately informed of the best available evidence and their decision is concordant with what matters most to them [11]. The complexities associated with identifying patient expectations are well documented and high quality shared decision-making requires the process to be patient-focused [8–12].

Exploring the goals that are most important to patients, whether those goals are likely to be achieved and how patients interpret their recovery will help health professionals to tailor their pre-surgical discussions. Through this process, health professionals may be able to temper expectations of surgery so that achievable goals can be set. Therefore, the primary aim of this study was to evaluate the improvement in quality of life in the context of achievement of individual patient goals, and the extent to which their pre-operative expectations were met. It was expected that the majority of goals related to pain, function and overall quality of life would be achieved and patients would be satisfied with how the TKA facilitated the achievement of their pre-operative expectations of these goals.

## Materials and methods

### Design and recruitment

This prospective cohort study followed a consecutive sample of 110 patients suffering from OA who were surveyed at baseline two weeks before receiving a primary TKA. The study was conducted from June 2019 till October 2020 at a multi-surgeon public hospital arthroplasty clinic. At one year, nine patients were unable to be followed up (three did not have surgery, six were not contactable), with no baseline difference between those who were lost to follow-up and the remaining cohort (Table 1). The sample was mostly female (*n* = 63, 57.3%), with a mean age of 71.8 ± 9.3 years.

**Table 1:** Participant demographics

	All patients (*n* = 110)	Eligible patients at one year (*n* = 101)	Ineligible patients at one year (*n* = 9)
Gender [n (%)]			
Female	63 (57.3)	57 (56.4)	3 (33.3)
Male	47 (42.7)	44 (43.6)	6 (66.7)
Age (years) [mean ± SD]	71.8 ± 9.3	72.0 ± 9.2	70.3 ± 10.4
TKA side [n (%)]			
Left	51 (46.4)	49 (48.5)	2 (22.2)
Right	49 (44.5)	43 (42.6)	6 (66.7)
Bilateral	10 (9.1)	9 (8.9)	1 (11.1)
SDM-Q-9 score [mean ± SD]	87.5 ± 13.5	87.4 ± 13.2	88.9 (17.5)
PCS [mean ± SD]			
Total score	27.6 ± 16.0	27.5 ± 15.9	28.9 ± 17.6
PCS rumination score	8.8 ± 5.6	8.7 ± 5.6	10.1 ± 6.1
PCS magnification score	5.9 ± 4.1	5.9 ± 4.1	5.7 ± 4.1
PCS helplessness score	12.9 ± 7.5	12.9 ± 7.5	13.1 ± 8.1
DQO Index [mean ± SD]	0.19 ± 0.18	0.20 ± 0.18	0.13 ± 0.11

*TKA* Total knee arthroplasty, *SDM-Q-9* Shared decision-making 9 item score (0 to 100, where a higher score indicates greater perceived shared decision-making), *PCS* Pain Catastrophising Scale (0 to 52, where a higher score indicates greater pain catastrophising), *PCS rumination* Thinking about how much the pain hurts (0 to 16, where a high score indicates greater pain focus), *PCS magnification* Amplification of the secondary effects of pain (0 to 12, where a higher score indicates greater amplification), *PCS helplessness* Concern for how much the pain affects overall life (0 to 24, where a higher score indicates greater concern), *DQO Index* Direct Questioning of Objectives (0 to 1, where a higher score indicates a higher level of quality of life), *SD* Standard deviation

At baseline, the degree to which patients felt they participated in shared decision-making with the health professionals treating them was assessed using the 9-item Shared Decision-Making Questionnaire (SDM-Q-9) scored out of 100 points (where a higher score indicated greater perceived shared decision-making) [13]. This patient-reported outcome measure asks questions about a patient’s experience with the decision-making process and measures the extent to which patients feel they are involved in the process of decision-making. Catastrophisation of pain was elicited using the Pain Catastrophising Scale (PCS) scored out of 52 points (where a higher score indicated greater pain catastrophisation) [14]. This patient-reported outcome measure has 13 items that ask the patient to reflect on past painful experiences and indicate the degree to which they agree with statements related to how they felt when in pain. The score provides three sub-scores; rumination (how much a patient thinks about pain), magnification (how much a patient worries about something serious happening due to the pain) and helplessness (how overwhelmed a patient feels due to the pain). The recruited sample reported participating in substantial shared decision-making (mean ± SD, 87.5 ± 13.5) and were assessed to report no overall pain catastrophisation (mean ± SD, 27.6 ± 16.0). However, these patients were identified to experience clinically meaningful magnification of the secondary effects of their pain according to the PCS magnification sub-score (Table 1).

Using the Direct Questioning of Objectives (DQO) Questionnaire [15–17] (Table 2), patients were interviewed by a single physiotherapist at baseline to identify their most important goals regarding their knee pain in context of the pending knee arthroplasty. Patients were asked to provide at least one and up to five of their most important goals of treatment. These were then collaboratively summarised into the patient’s own words so they could be provided to the patient to respond to one year after their surgery [18]. The DQO Questionnaire included the patient’s assessment of two domains for each goal on 11-point numerical rating scales: (1) the importance of the goal; and (2) the current ability to achieve the goal. For the purpose of this study, the reported values for importance and ability were interpreted as low if less than or equal to five, and high if greater than or equal to eight. Participants were also asked to assess their expectation that a TKA would help achieve the goal on an 11-point numerical rating scale. Patient goals were categorised into 15 themes and then condensed into 10 major categories to facilitate analysis of the key concepts raised by patients. This process was conducted by three authors using an inductive thematic approach in pursuit of saturation of themes raised by patients (Tables 3, 4 and 5). At one year after receiving a TKA, individual goals (stated pre-operatively) were provided back to the patient, when they were asked to rate their current ability and extent to which they were satisfied with how the knee arthroplasty helped achieve their expected goals, in absence of their pre-operative assessment of expectations of those goals. This was scored on a similar 11-point numerical rating scale as the baseline DQO survey. A quality of life index (DQO Index) on a scale of 0 to 1 was calculated for each major category and then overall. This was calculated at baseline and follow-up using the product of each goal’s importance at baseline and ability at baseline and follow-up, respectively [15–17] (Table 2). For the purposes of this study, quality of life using the DQO Index was interpreted as poor (≤ 0.25), moderately poor (0.26–0.49), moderately high (0.50–0.74) and high (≥ 0.75). Where multiple goals within the same category were reported at baseline (e.g., walking ability affected by pain), the goal with the highest importance was retained for final scoring. When this was not able to differentiate a single clear goal to use in the calculation (i.e., identical importance given to two or more goals within one theme), then a process of elimination was employed until there was a single remaining goal. In this process of elimination, the first criterion was the goal with the lowest baseline ability. If two or more remaining goals had the same ability, then the goal with the highest expectation was chosen. After this the goal with the lowest reported ability at one year and finally the lowest estimation of contribution of the knee arthroplasty to addressing their goal at one year, was chosen.

**Table 2:** Example of calculation of the Direct Questioning of Objectives Index

	Patient objective	Importance of objective (scale 0 to 10)	Ability to achieve objective (scale 0 to 10)	Product of previous 2 columns divided by 10
1	Be more mobile	10	5	5.0
2	Going up 10 steps	8	0	0.0
3	Mowing the lawn	7	4	2.8
Total column sum		25		7.8

Direct Questioning of Objectives Index (weighted mean) = 7.8 ÷ 25 = 0.31 (scale from 0 to 1, where a higher score indicates higher attainment of quality of life objectives)

**Table 3:** Themes of goals identified

	Themes	Unique patients [*n* (%)]	Frequency [*n*]	Importance/10 [mean ± SD]	Initial ability/10 [mean ± SD]	Expectation/10 [mean ± SD]
1	Reduce pain in the knee	101 (91.8)	270	9.5 ± 1.0	1.6 ± 2.1	9.3 ± 1.1
2	Improve ability to mobilise	101 (91.8)	124	9.2 ± 1.3	2.6 ± 2.5	9.0 ± 1.3
3	Improve function in daily tasks	89 (80.9)	144	9.5 ± 1.0	1.7 ± 2.1	9.3 ± 1.1
4	Negotiating altered terrain (stairs, hills, uneven surfaces)	69 (62.7)	83	9.3 ± 1.1	2.1 ± 2.4	9.0 ± 1.2
5	Improve mobility of knee	43 (39.1)	58	9.3 ± 1.3	2.5 ± 2.2	8.9 ± 1.2
6	Improve function in recreational physical activity	41 (37.3)	54	9.3 ± 1.2	2.3 ± 2.6	9.4 ± 1.1
7	Improve quality of life	31 (28.2)	35	9.8 ± 0.7	2.8 ± 2.7	9.2 ± 1.0
8	Being able to get down and up from the floor	31 (28.2)	34	9.6 ± 0.9	2.2 ± 2.4	8.9 ± 1.1
9	Being able to participate in social activities	30 (27.3)	35	9.2 ± 1.4	2.5 ± 2.4	8.7 ± 1.5
10	Reducing fatigue and improving sleep	27 (24.6)	27	9.9 ± 0.5	1.3 ± 1.8	9.1 ± 1.1
11	Negotiating thresholds	18 (16.4)	19	8.6 ± 1.5	1.8 ± 2.2	8.7 ± 1.5
12	Being able to return to work	11 (10.0)	13	9.8 ± 0.6	2.6 ± 1.8	9.3 ± 1.0
13	Preventing falls	10 (9.1)	10	9.5 ± 1.1	2.4 ± 2.2	9.0 ± 1.2
14	Being able to participate in hobbies	8 (7.3)	8	8.5 ± 1.9	3.0 ± 1.6	8.8 ± 1.6
15	Reduce issues in areas other than the knee	5 (4.6)	5	9.6 ± 0.9	2.6 ± 2.8	8.6 ± 1.3

Importance (scale from 0 to 10, where 0 = not important and 10 = very important); Ability (scale from 0 to 10, where 0 = no ability and 10 = complete ability; Expectation of TKA (scale from 0 to 10, where 0 = not address and 10 = completely address)

*n* Number, *SD* Standard deviation

**Table 4:** Follow-up of Direct Questioning of Objectives Index

	Category*	Timepoint	DQO [mean ± SD]	Mean difference [mean ± SD]	95%CI	*p* value
1	Mobility (*n* = 97)	Baseline	0.17 ± 0.23	0.50 ± 0.08	0.43–0.58	< 0.001
		1 year follow-up	0.66 ± 0.31			
2	Reduce pain in knee (*n* = 93)	Baseline	0.14 ± 0.21	0.58 ± 0.07	0.51–0.65	< 0.001
		1 year follow-up	0.73 ± 0.29			
3	Improve daily tasks (*n* = 82)	Baseline	0.25 ± 0.26	0.45 ± 0.08	0.38–0.53	< 0.001
		1 year follow-up	0.70 ± 0.27			
4	Social & hobbies (*n* = 55)	Baseline	0.26 ± 0.27	0.44 ± 0.10	0.35–0.53	< 0.001
		1 year follow-up	0.70 ± 0.27			
5	Knee range of motion (*n* = 39)	Baseline	0.21 ± 0.21	0.53 ± 0.09	0.45–0.61	< 0.001
		1 year follow-up	0.74 ± 0.19			
6	Improve quality of life (*n* = 29)	Baseline	0.27 ± 0.26	0.54 ± 0.13	0.41–0.68	< 0.001
		1 year follow-up	0.81 ± 0.25			
7	Fatigue & sleep deprivation (*n* = 24)	Baseline	0.13 ± 0.18	0.57 ± 0.13	0.44–0.71	< 0.001
		1 year follow-up	0.70 ± 0.27			
8	Returning to work (*n* = 10)	Baseline	0.27 ± 0.21	0.47 ± 0.16	0.26–0.67	0.001
		1 year follow-up	0.73 ± 0.16			
9	Falls prevention (*n* = 9)	Baseline	0.24 ± 0.22	0.44 ± 0.22	0.19–0.69	0.003
		1 year follow-up	0.68 ± 0.26			
10	Reduce “non-knee” related medical issues (*n* = 5)	Baseline	0.26 ± 0.28	0.46 ± 0.31	0.06–0.86	0.033
		1 year follow-up	0.72 ± 0.22			
	Overall	Baseline	0.20 ± 0.18	0.52 ± 0.05	0.46–0.57	< 0.001
		1 year follow-up	0.71 ± 0.21			

*DQO* Direct Questioning of Objectives Index (scale from 0 to 1, where a higher score indicates higher attainment of quality of life goals)

*n = 101 patients who completed 1 year follow-up

**Table 5:** Major categories derived from goals

	Category	No. unique patients [*n* (%)]	Frequency [*n*]	Importance/10 [mean ± SD]	Initial ability/10 [mean ± SD]	Ability at 1 year*/10 [mean ± SD]	Mean difference in ability [mean (95%CI)]	Initial expectation/10 [mean ± SD]	Achievement of expectations at 1 year*/10 [mean ± SD]	Mean difference in expectation [mean (95%CI)]
1	Mobility (goal 2, 4, 8, 11)	106 (96.4)	260	9.8 ± 0.5	1.6 ± 2.3	6.6 ± 3.1	5.0 (4.3–5.7)^‡^	9.3 ± 1.2	7.1 ± 3.1	−2.2 (−2.8– −1.6)^‡^
2	Reduce pain in knee (goal 1)	101 (91.8)	270	9.8 ± 0.6	1.4 ± 2.1	7.3 ± 2.9	5.9 (5.2–6.6)^‡^	9.5 ± 1.0	7.6 ± 2.7	−1.9 (−2.4– −1.4)^‡^
3	Improve daily tasks (goal 3)	89 (80.9)	144	9.6 ± 0.8	2.4 ± 2.5	7.0 ± 2.7	4.6 (3.9–5.3)^‡^	9.1 ± 1.2	7.4 ± 2.8	−1.7 (−2.3– −1.1)^‡^
4	Social & hobbies (goal 6, 9, 14)	60 (54.6)	97	9.4 ± 1.1	2.5 ± 2.7	7.0 ± 2.7	4.5 (3.8–5.2)^‡^	9.2 ± 1.1	7.5 ± 2.6	−1.7 (−2.2– −1.2)^‡^
5	Knee range of motion (goal 5)	43 (39.1)	58	9.2 ± 1.3	2.1 ± 2.1	7.4 ± 1.9	5.3 (4.8–5.8)^‡^	8.9 ± 1.2	7.7 ± 2.2	−1.2 (−1.7– −0.7)^‡^
6	Improve quality of life (goal 7)	31 (28.2)	35	9.7 ± 0.7	2.6 ± 2.6	8.1 ± 2.5	5.5 (4.8–6.2)^‡^	9.3 ± 1.0	8.4 ± 2.4	−0.9 (−1.4– −0.4)^‡^
7	Fatigue & sleep deprivation (goal 10)	27 (24.6)	27	9.9 ± 0.5	1.3 ± 1.8	7.0 ± 2.7	5.7 (5.1–6.3)^‡^	9.1 ± 1.1	7.2 ± 2.9	−1.9 (−2.5– −1.3)^‡^
8	Returning to work (goal 12)	11 (10.0)	13	9.7 ± 0.7	2.6 ± 1.9	6.8 ± 1.6	4.2 (3.7–4.7)^‡^	9.5 ± 0.9	6.9 ± 2.1	−2.6 (−3.0– −2.2)^‡^
9	Falls prevention (goal 13)	10 (9.1)	10	9.5 ± 1.1	2.4 ± 2.2	7.3 ± 2.6	4.9 (4.3–5.6)^‡^	9.0 ± 1.2	7.8 ± 3.4	−1.2 (−1.9– −0.5)^‡^
10	Reduce “non-knee” related medical issues (goal 15)	5 (4.6)	5	9.6 ± 0.9	2.6 ± 2.8	7.2 ± 2.2	4.6 (3.9–5.3)^‡^	8.6 ± 1.3	8.0 ± 2.5	−0.6 (−1.1– −0.1)^†^

Importance (scale from 0 to 10, where 0 = not important and 10 = very important); Ability (scale from 0 to 10, where 0 = no ability and 10 = complete ability; Expectation of TKA (scale from 0 to 10, where 0 = not address and 10 = completely address); Achievement of expectations due to TKA (0 = not to satisfaction and 10 = completely to satisfaction)

*n* Number, *SD* Standard deviation, *95%CI* 95% confidence interval

*Note improvement in ability score is for *n* = 101 patients who completed 1 year follow-up

^†^*p* ≤ 0.050

^‡^*p* ≤ 0.001

This study was reported as recommended by the STROBE Initiative reporting guidelines (Appendix A) [19].

### Statistical analysis/Power

To the best of our knowledge, this is the first time the DQO questionnaire has been used in this population and therefore there are no comparative papers to establish an orthopaedic specific sample size calculation. Based on previous studies investigating the clinically meaningful changes to the DQO in patients undergoing surgery in non-orthopaedic populations [16, 17], a sample size of 36 was required to achieve 80% power to detect a mean paired difference of 0.211 with an estimated standard deviation of differences of 0.433 and with a significance (α) of 0.05 using a two-sided paired *t*-test [20, 21].

All statistical calculations were conducted using SPSS version 27. Patient, goal and objective category characteristics were analysed descriptively, with categorical data presented as frequencies (percentage), and continuous data as mean and standard deviations (SD). Differences in distribution for continuous data were analysed using an independent samples *t*-test or Mann-Whitney U test based on normalcy of distribution. Differences in distribution for categorical data was analysed using a chi square or Fisher’s exact test. A *p* < 0.05 was considered statistically significant for all performed analyses.

## Results

### Patient goals

When looking at the 15 themes reported initially by patients, the main goals focused on ‘reducing pain in the knee’ (*n* = 101 patients; 91.8%), ‘improving the ability to mobilise’ (*n* = 101 patients; 91.8%), ‘improving function in activities of daily life’ (*n* = 89 patients; 80.9%) and ‘being able to negotiate altered terrain’ (*n* = 69 patients; 62.7%) (Table 3). Whilst the themes of ‘reducing pain in the knee’ and ‘improving the ability to mobilise’ were reported by the same number of patients, the frequency of individual goals was much higher for ‘reducing pain in the knee’. Patients reported ‘improving function in activities of daily life’ slightly more often than the goal to ‘improve the ability to mobilise’. Reports of other themes were much lower, with themes such as ‘improving quality of life’ (*n* = 31 patients; 28.2%) and ‘reducing fatigue & improving sleep’ (*n* = 27 patients; 24.6%) not being identified as commonly. For all themes, importance of the goal and expectations that a knee arthroplasty would help achieve the goal were high, whilst current ability to achieve the goal was low.

On categorisation of the overall major goals at baseline, most patients identified ‘mobility’ (*n* = 106 patients; 96.4%), ‘reducing pain in the knee’ (*n* = 101 patients; 91.8%), ‘improving daily tasks’ (*n* = 89 patients; 80.9%) and ‘re-engaging with social & hobby activities’ (n = 60 patients; 54.6%) as their goals of treatment (Tables 4 and 5). When collaborating four initial themes into the category of ‘mobility’, the frequency of being reported increased (n = 260 times reported) to much closer to that of ‘reducing pain in the knee’ (n = 270 times reported). At baseline, patients were found to have an overall poor quality of life according to the DQO Index (mean ± standard deviation: 0.19 ± 0.18), reflecting a poor ability to achieve their desired goals (Table 1).

### Follow-up at one year

A moderately large change in the overall improvement in disease-specific, health-related quality of life using the DQO Index, in context of patient-reported achievement of individual patient goals, was found at one year follow-up (mean difference ± standard deviation: 0.52 ± 0.05) (Table 4). The improvements per category in the DQO Index ranged from 0.44 to 0.58, with the largest changes in the categories of ‘reducing pain in the knee’ (mean difference ± standard deviation: 0.58 ± 0.07), ‘improving fatigue & sleep deprivation’ (mean difference ± standard deviation: 0.57 ± 0.13), ‘improving quality of life’ (mean difference ± standard deviation: 0.54 ± 0.13) and ‘improving knee range of motion’ (mean difference ± standard deviation: 0.53 ± 0.09) (Table 4).

Patients generally reported large improvements in their ability to achieve their goals (Table 5). The categories with largest improvements in ability reflected those identified in the DQO Index when evaluating the degree of change to quality of life. The smallest improvements in ability to achieve goals were seen in ‘improving daily tasks’, ‘reduction of non-knee related medical issues’, ‘re-engagement with social & hobby activities’, and ‘returning to work’. Despite improvements in ability across all categories of goals at one year post-operatively (range 4.2 to 5.9), patients reported lower achievement of these goals relative to their pre-operative expectations (mean difference range: 0.6 to 2.6 on an 11-point scale) (Table 5). The largest differences between pre-operative expectation that a TKA would help achieve each goal and the patient’s belief of the extent to which the TKA helped achieve their goal one year after knee arthroplasty was found in ‘ability to return to work’ (mean difference: −2.6, 95%CI −3.0 to −2.2), ‘improvement in mobility’ (mean difference: −2.2, 95%CI −2.8 to −1.6), ‘improving fatigue & sleep deprivation’ (mean difference: −1.9, 95%CI −2.5 to −1.3), and ‘reduction of pain in the knee’ (mean difference: −1.9, 95%CI −2.4 to −1.4). The smallest differences were found in ‘reduction of non-knee related medical issues’ (mean difference: −0.6, 95%CI −1.1 to −0.1), ‘improving quality of life’ (mean difference: −0.9, 95%CI −1.4 to −0.4), ‘improving knee range of motion’ (mean difference: −1.2, 95%CI −1.7 to −0.7) and ‘prevention of falls’ (mean difference: −1.2, 95%CI −1.9 to −0.5) (Table 5).

## Discussion

### Statement of principal findings

This study investigated the effect of knee arthroplasty on quality of life, in context of the goals patients have pre-operatively. Further, this study evaluated the extent to which these pre-operative expectations were met, one year after undergoing a TKA. A moderately large change in overall quality of life was identified using the DQO Index. The largest post-operative improvements in quality of life per patient-reported category were found in ‘reducing pain in the knee’, ‘improving fatigue & sleep deprivation’, ‘improving quality of life’ and ‘improving knee range of motion’. Whilst pre-operatively believing a TKA would help them improve their ability to achieve their goals by a large amount and ultimately achieve their goals, patients reported not often accomplishing their goals to the degree of their pre-operative expectations.

### Meaning of the study

This study suggests focal points for health professionals to use when guiding patients through a high quality decision-making process. Despite patients pre-operatively reporting participating in substantial shared decision-making, their pre-operative goals were not achieved to the extent they expected. This finding suggests that the information patients were provided when making their decision may not have addressed their specific goals, or adequately moderated expectations. In line with previous research, the key points likely to be required for discussion include patient’s expectations of addressing their pain in the knee, improvements in mobility and sleep related issues [7, 9, 22]. Multiple other individual themes and categories for goals were identified, which could be incorporated into routine discussions with patients to help establish realistic expectations of post-operative outcomes. The findings of this study also showed lower than expected post-operative patient-reported quality of life assessed through the DQO Index (a change from poor pre-operatively to moderately high one year post-operatively), in context of relatively large differences between pre-operative expectations of surgery and post-operative achievement of goals. This supports previous research that has recommended improvements in the implementation of personalised treatment plans [23–25]. By improving shared decision-making related to whether TKA is the best option for an individual patient considering their unique expectations and clinical presentation, patients may be able to recognise that there is likely variation in the way that their arthritis presents or responds to the implantation of a knee prosthesis, identify what is important to them and align their views to realistic outcomes of this surgery [26–29].

### Strengths and weaknesses of the study

There has been great interest in eliciting the pre-operative needs and expectations of patients in this population, especially if these have been adequately addressed or achieved at longer term follow-up [7–9, 22]. This study took a novel approach to evaluating the achievement of patient goals by using a quality of life index that incorporated pre-operative importance and ability to achieve unique goals, to post-operative ability and achievement of these goals. This is a novel outcome measure that has been applied in the surgical setting previously. Though, cut-off scores for its use in this population have not been evaluated and interpretations in the current study were based on expert opinion and consensus amongst the authors. The study was also adequately powered to evaluate the stated aims and was similar in size to a similar study by Lange et al. [22]. However, this is the first time that the DQO Index has been used in an orthopaedic population, and therefore the degree of change in scores should be interpreted with caution.

The study employed a prospective design which included the open-ended interviewing of individual patients by a qualified health professional directly before undertaking a TKA. Goals were also pursued until saturation using an inductive thematic approach, confirmed between three authors. This approach allowed for exploration of key themes of interest to each patient. Through collaboratively summarising goals into the patient’s own words, these responses were better focused to what patients believed TKA would help them achieve and therefore may provide a more accurate representation of the importance and expectations of fulfilment for each goal. Other methodologies focussing on assessing this highly granular concept may not have the same ability to represent the views and complexities of individual patients [8, 9, 23]. A further strength of this study was the use of a previously employed analytical approach used in surgical settings to identify the achievement of pre-operative goals [15–17].

Despite the strengths of this study, there are limitations that should be considered. While eliciting data qualitatively can provide a rich source of information, it is by definition exposed to confirmation bias and variation in interpretation due to its openness [30]. This was attempted to be mitigated through having all themes reviewed and confirmed by multiple authors familiar with treating this patient population, prior to being categorised. Another potential limitation of this study was the inclusion criteria of patients suffering from OA in a single country. Though this implies that results may limit the scope of this study, it may be argued that most patients receiving TKA present with OA as their primary diagnosis and previous studies conducted in various countries have generally identified similar types of goals reported in the current study [7, 9, 23–25, 29, 31].

### Unanswered questions and future research

This study contributed to the evidence that patients and health professionals need to work towards a more cohesive communication strategy regarding the outcomes of TKA. Research surrounding what patients want to achieve and how health professionals process this information when proposing a treatment plan may assist in understanding where breakdowns in setting realistic expectations occur. This is further challenged where health professionals attempt to apply treatments that have worked in past without considering the unique needs of each patient or where patients are unable to convey their views effectively, particularly found in patients of lower health literacy [32]. Considering individual health professionals have unique approaches to improving the quality of life of their patients, it is important for health professionals to reflect on their own biases, communicate effectively between each other, and aim to elicit consistent information about the issues that each patient is facing or goals they want to achieve [33–36]. This is integral to the decision-making process wherein patients may receive inconsistent recommendations and warrants further investigation regarding the implementation of suggested models [37].

Investigation of the optimal process for transferring information and the communication style required to convey realistic expectations should be supported. Previous studies have attempted to generate patient-focused outcome measures that incorporate generic goals patients want addressed. These measures try to facilitate health professionals to focus on eliciting what patients want to achieve and how the outcomes of these may be assessed [7, 23]. A significant body of work has been established to ensure patients are able to use these effectively, informing health professionals of what needs to be focused on. However, continued exploration of the utility of these outcome measures is required to ensure they can be effectively interpreted by patients and align with the required information to inform the decision-making process [4, 38, 39].

This study supports the established idea that healthcare professionals should be guiding their patients to communicate their expectations to ensure the best possible outcome for themselves. While there may be many approaches to this, ensuring patients are engaged with the shared decision-making process and their goals are uniquely reflected in their choices is paramount to the recommendation of the best available care [40]. In this way, a tailored education process with a structured education schedule incorporating the key goals and expectations of patients may be warranted. Development of materials, such as patient decision-aids, may be key in the success of these activities [34, 40, 41].

## Conclusions

This study found patients undergoing TKA do not achieve most of their unique goals of treatment one year after surgery, to the extent of their pre-operative expectations. This was particularly found in the goals of improving pain in the knee, mobility, sleep related activities and ability to return to work. Future studies should further evaluate the decision-making process and options to educate patients on realistic expectations of surgery to facilitate an optimised informed decision-making pathway.

### Electronic supplementary material

Below is the link to the electronic supplementary material.

Supplementary Material 1

## Data Availability

The data that support the findings of this study are not openly available due to reasons of sensitivity and are available from the corresponding author upon reasonable request. Data are located in controlled access data storage at Royal Prince Alfred Hospital, Camperdown, New South Wales, Australia.
